# Near-infrared fluorescent clip guided gastrectomy: Report of 2 cases (Case reports)

**DOI:** 10.1016/j.amsu.2020.04.026

**Published:** 2020-05-11

**Authors:** Satoshi Narihiro, Masashi Yoshida, Hironori Ohdaira, Hideyuki Takeuchi, Teppei Kamada, Rui Marukuchi, Norihiko Suzuki, Sojun Hoshimoto, Takayuki Sato, Yutaka Suzuki

**Affiliations:** a Department of Surgery, International University of Health and Welfare Hospital, 537-3, Iguchi, Nasushiobara City, Tochigi, 329-2763, Japan; b Center for Photodynamic Medicine, Kochi University, Kohasu Oko-cho 185-1, Nankoku, Kochi, 783-8505, Japan

**Keywords:** Gastric cancer, Fluorescent clip, Intraoperative endoscopy, NIRF, Near-infrared fluorescent

## Abstract

**Introduction:**

This is the first report on near-infrared fluorescent (NIRF) clip-guided gastrectomy. The NIRF clip, ZEOCLIP FS, emits NIRF signals when excited. We hypothesized that preoperative placement of the ZEOCLIP FS near a gastric lesion would allow fluorescence laparoscopic localization of the clip, and hence, the lesion, during surgery. We report this technique in two cases.

**Case presentation:**

Case 1: An 81-year-old female was diagnosed with early gastric cancer and a pedunculated 4 cm large hyperplastic polyp that had prolapsed into the duodenum, and was scheduled for laparoscopy-assisted distal gastrectomy, due to the potential risk of dissection of the polyp with the duodenal wall. On the day before surgery, ZEOCLIP FS clips were endoscopically placed at the cancer site and the polyp. The locations of the fluorescent clips were confirmed intraoperatively using a full-color fluorescence laparoscope.

**Case 2:**

An 81-year-old male was scheduled for laparoscopy-assisted total gastrectomy for gastric cancer under fluorescent clip-guidance. Clip locations could not be confirmed during initial intraoperative observation. However, when the stomach wall was raised using forceps during a second observation attempt, the fluorescent clip locations were confirmed.

**Discussion:**

In case 1, it was easy to confirm clip location, facilitating complete resection of early gastric cancer without dissecting the polyp. In case 2, the fluorescent clip was located by raising the stomach and adjusting the angle between the stomach wall and the fluorescence laparoscope.

**Conclusion:**

The positive results of these two cases warrant conducting feasibility studies for use of this method.

## Highlights

• This is the first case series on near-infrared fluorescent clip guided gastrectomy.
• It was possible to observe the position by devising the angle between the stomach wall and the fluorescent laparoscope.
• The near-infrared clip method may be useful as a method for confirming an intraoperative lesion site in gastrectomy.

## 1 Introduction

These case reports have been reported in line with the SCARE criteria [1]. Near-infrared fluorescent (NIRF) radiation penetrates soft tissues [[2], [3], [4]] and can be detected as translucent light through the wall of hollow organs when both fluorescence and excitation sources have a NIR wavelength and are observed with a NIRF camera [5]. Since 2016, we have been cooperating in developing NIRF clips for tumor site marking. The NIRF clip, ZEOCLIP FS, with peak excitation and fluorescence wavelengths of 760 and 790 nm, were manufactured and approved for clinical use in 2019 (Reg. No. 13B1X001111000020). The ZEOCLIP FS has a fluorophore resin-filled tip, which emits NIRF signals when excited. During laparoscopic gastrectomy, intraoperative confirmation of the lesion before it is resected is important. The method used for intraoperative lesion confirmation and to determine the resection margin is one of the factors determining oncological safety of the procedure and whether it is possible to achieve function preserving gastrectomy [6]. Therefore, tumor site marking before laparoscopic gastrectomy is becoming increasingly important.

We hypothesized that preoperative intraluminal placement of the ZEOCLIP FS near the lesion, and its subsequent intraoperative observation using a fluorescence laparoscope during surgery would facilitate localization of the lesion. We first started using the clip in colorectal cancer cases, and after confirming a certain level of effectiveness [7], started using it in cases involving the stomach. Near-infrared light reportedly penetrates soft tissues up to a thickness of 10 mm [[8], [9], [10]]. Hence, we were not sure whether clips positioned in the stomach could be visualized using fluorescence laparoscopy, because the thickness of the stomach wall is about 8 mm, which is thicker than that of the colon wall [11]. This is the first report of NIRF clip-guided gastrectomy. We used the procedure in two eligible cases. This study was approved by the Institutional Review Board of the authors’ institution (No. 13-B-344).

## 2 Presentation of cases

**Fig. 1:**
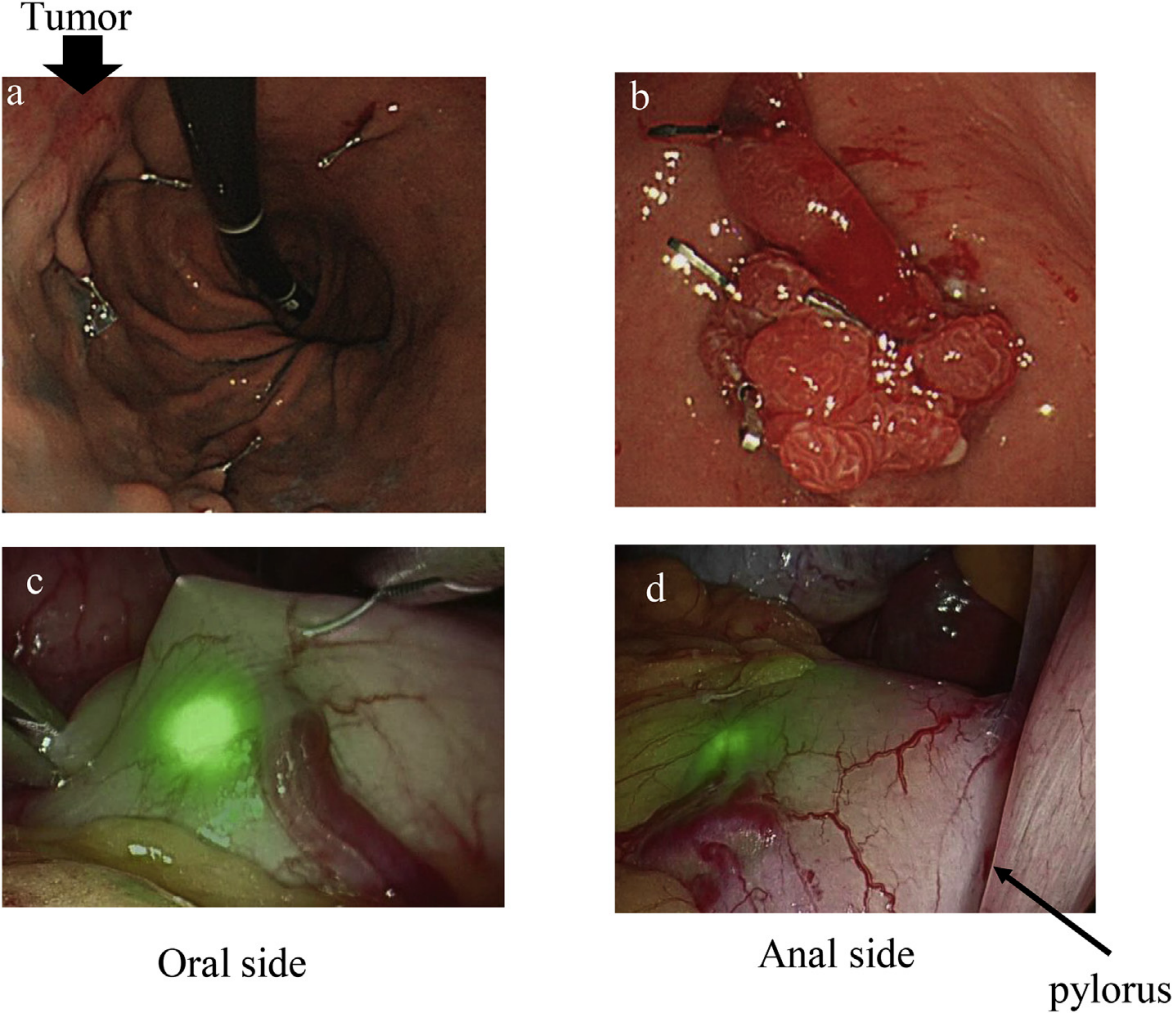
Preoperative endoscopic and intraoperative laparoscopic view of the clips in Case 1. a) b) Endoscopic view: Clips were intraluminally placed around the gastric lesion, two on the proximal side of the tumor, one clip each on the small and large bowel side, and four at the polyp site, respectively. C) d) Laparoscopic view: Locations of the fluorescent clips were intraoperatively confirmed using a full-color fluorescence laparoscope, VisionSense (Medtronic, MN, USA). (For interpretation of the references to color in this figure legend, the reader is referred to the Web version of this article.)

Case 1: An 81-year-old female presented to our hospital for gastric cancer screening. She was diagnosed with early gastric cancer (M, post, cType0-IIc, cT1b, cN0, M0) and a 4 cm large, pedunculated, hyperplastic gastric polyp that had prolapsed into the duodenum. She was scheduled for laparoscopy-assisted distal gastrectomy, which had the potential risk of dissecting the polyp with the duodenal wall. The patient's consent was obtained for intraoperative use of the newly designed fluorescent clip and for reporting the study results. On the day preceding surgery, during upper endoscopy, ZEOCLIP FS were placed at two sites because removal of both the tumor and the polyp was planned. Two clips were deployed on the proximal side of the tumor, with one clip each placed on the lesser and greater curvatures, at the same level as the tumor. Three clips were deployed on the polyp and one clip was deployed on its peduncle (Fig. 1a and b). During surgery performed the following day, the locations of the fluorescent clips were confirmed using a full-color fluorescence laparoscope, Vision Sense® (Medtronic, Minneapolis, MN, USA) (Fig. 1c and d). Vision Sense is a new near-infrared fluorescence laparoscope that allows bright field full color observation, and has the property that it can adjust the intensity of excitation light and quantify the intensity of fluorescence during observation. Using the clips as a guide, we were able to successfully identify the appropriate proximal and distal resection margins, and were able to remove the tumor and polyp as a single specimen. Pathological evaluation confirmed the preoperative findings (tumor: pT1b, pN0, pM0; polyp: Hyperplastic polyp). There were no adverse events associated with use of the clips. The patient's postoperative course was uneventful and she was discharged on the seventh postoperative day.

**Fig. 2:**
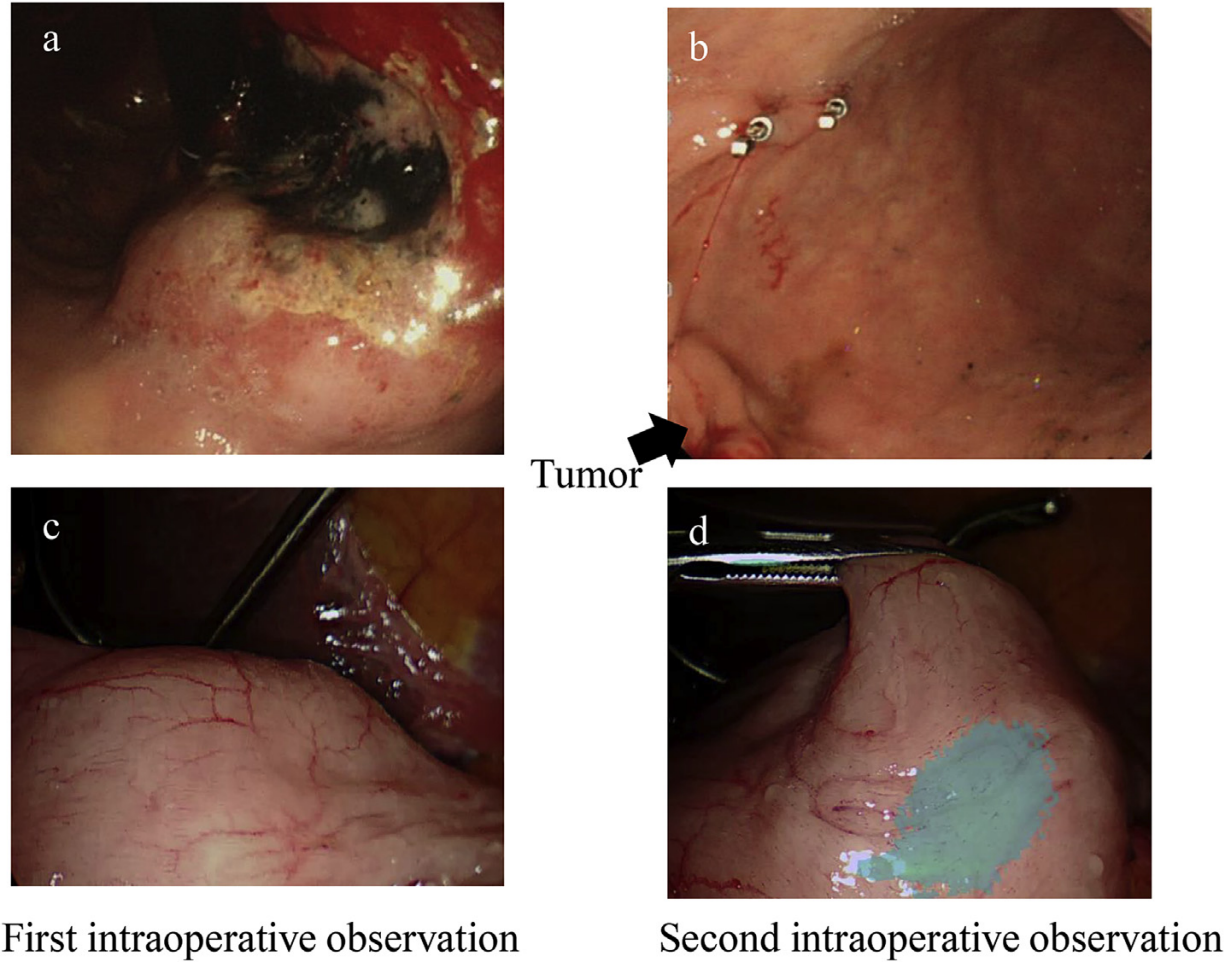
Preoperative endoscopic and intraoperative laparoscopic view of the clips in Case 2. a) Endoscopic view of the gastric cancer (U, post, cType2, cT3, cN0, M0). B) Two clips were placed intraluminally around the tumor on the distal side of the tumor, as seen endoscopically. C) The locations of the fluorescent clips could not be confirmed during initial laparoscopic observation. D) Subsequently, when the stomach wall was raised and the excitation light was applied perpendicular to the gastric wall during a second intraoperative observation, the locations of the fluorescent clips were confirmed.

Case 2: An 81-year-old male presented to our hospital with anemia. He was diagnosed with gastric cancer (U, post, cType2, cT3, cN0, M0) (Fig. 2a) and was scheduled for laparoscopy-assisted total gastrectomy. Preoperatively, under upper endoscopy, two ZEOCLIP FS clips were placed intraluminally around the cancerous lesion on the distal side of the tumor (Fig. 2b). Intraoperatively, during the initial observation, we could not confirm the locations of the fluorescent clips (Fig. 2c). Subsequently, however, by raising the stomach wall and applying the excitation light perpendicular to the gastric wall during a second attempt at intraoperative observation, we were able to confirm the locations of the fluorescent clips (Fig. 2d). In this case, although ZEOCLIP FS clips were placed in the field of view for the possibility of distal gastrectomy, when we confirmed the location of the clips using Vision Sense during surgery, it was determined that the tumor was indicated for total gastrectomy. Pathological evaluation of the resected specimen confirmed the preoperative findings (pT3, pN0, pM0). As with the previous case, there were no adverse events associated with use of the clips. The patient's postoperative course was uneventful and he was discharged on the seventh postoperative day.

## 3 Discussion

In case 1, using the clips as a guide, it was easy to confirm the tumor location in the stomach, which facilitated intraoperative determination of the proximal resection margin. The position of the prolapsed polyp could also be clearly identified, facilitating determination of the distal resection margin in the duodenum.

**Fig. 3:**
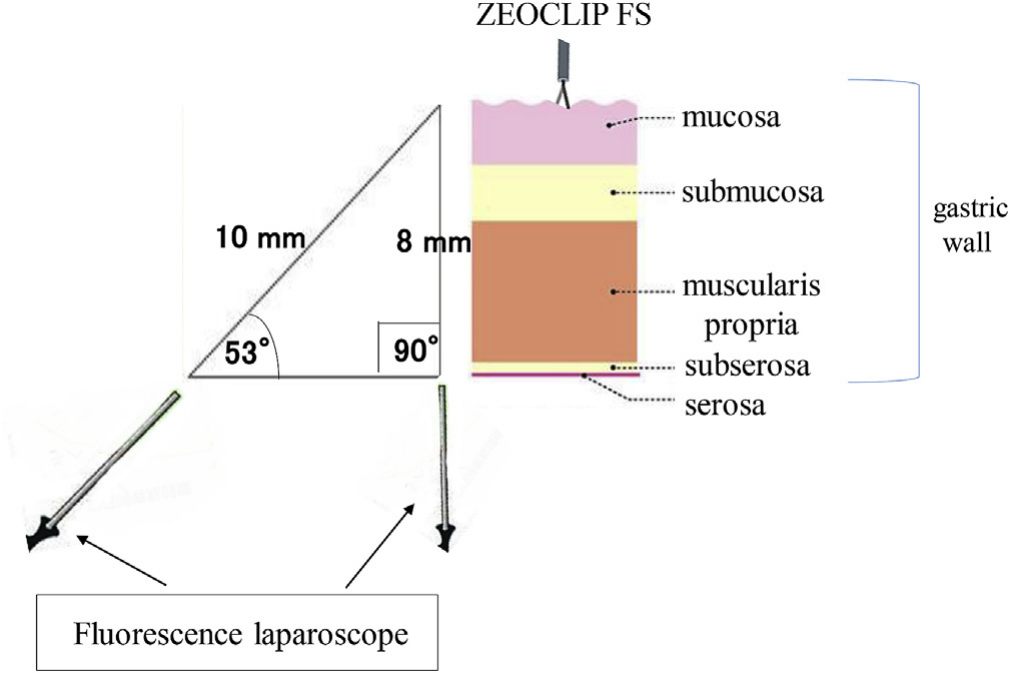
Schematic diagram showing the relationship between the angle between the laparoscope and gastric wall and transmission distance of the near-infrared light. It is estimated that the laparoscope should be at an angle of 53 or more to the stomach wall for application of the excitation light to the clip.

In case 2, contrivance was needed for observation of the fluorescence. Based on our experience with fluorescence-guided surgery for colorectal cancer using ZEOCLIP FS, we adjusted the viewing angle of the fluorescence laparoscope relative to that of the gastric wall to confirm the locations of the fluorescent clips using a full-color fluorescence laparoscope during surgery. In the first attempt at intraoperative clip observation using the fluorescence laparoscope, the locations of the fluorescent clips were not identifiable because we did not pay attention to the angle of the fluorescence laparoscope relative to the gastric wall. However, by raising the stomach using forceps, we were able to confirm the locations of the fluorescent clips. Raising the stomach might have changed the angle between the stomach wall and the fluorescence laparoscope. Ideally, the transmission distance through the stomach wall of the excitation light emitted by the fluorescence laparoscope should be as short as possible. Assuming the stomach wall is 8 mm thick, when the fluorescence laparoscope is placed perpendicular to the stomach, the excitation light should be able to identify the clip through the 8 mm wall. Placing the fluorescence laparoscope at an angle to the stomach wall increases the transmission distance. For example, if the laparoscope is placed at an angle of 53° to the stomach wall, the transmission distance is 10 mm. If the transmission distance through the stomach wall exceeds 10 mm, the fluorophore tip of the clip is not excited, and hence, the location of the clip might not be recognized. This suggests that the angle of the laparoscope relative to the stomach wall should be 53° or more to allow appropriate application of the excitation light to the clip (Fig. 3). Raising the stomach wall using forceps might not only change the viewing angle between the fluorescence laparoscope and stomach wall, but also reduce the thickness of the stomach wall by an extension force, thus shortening the transmission distance. The focal intensity of light is also affected by the transmission distance, which alters the color of the fluorescence, as seen in the difference in fluorescence between the images for case 1 and case 2. In our previous cases of preoperative marking of the tumor site in the large intestine using the clips, four fluorescent clips were placed intraluminally at angles of 90° around the lesion. However, this method is technically difficult in the stomach. We believe that for lesions of the stomach, it is important to place the clips on the greater curvature and/or the anterior stomach wall to enable confirmation of the location of the fluorescent clips. Details about the number of clips to be placed to confirm the exact type of surgery needed for complete resection of the tumor should be assessed in future studies. More cases of fluorescent clip-guided gastrectomy need to be performed to confirm the utility.

Currently, intraoperative endoscopy is usually used for intraoperative localization of gastric tumors, although the procedure has several drawbacks, such as prolonged operation time and need of a skilled endoscopist [[12], [13], [14]]. Intraoperative fluorescent clip-guided tumor marking is a simple and inexpensive alternative (the approximate cost per clip is $100, with no additional insertion charge), to intraoperative endoscopy. If the effectiveness of fluorescent clip-guided gastrectomy is confirmed, tumor marking with the ZEOCLIP FS has the potential to replace intraoperative endoscopy as a method of intraoperative localization of gastric tumors.

## 4 Conclusion

This is the first report of fluorescent clip-guided gastrectomy. In our patients, preoperative endoscopic placement of the fluorescent clips was easy and was performed without any adverse events. Insertion of the clips did not prolong the duration of endoscopy. Intraoperatively, it was possible to observe the position of the clips by adjusting the angle between the stomach wall and the fluorescence laparoscope. The near-infrared clip method might be useful for intraoperative confirmation of the tumor site during gastrectomy.

## Author contribution

SN have made substantial contributions to conception and design, or acquisition of data, or analysis and interpretation of data.

MY have been involved in drafting the manuscript or revising it critically for important intellectual content.

HT have get in on a discussion about this study.

TK have get in on a discussion about this study.

RM have get in on a discussion about this study.

NS have get in on a discussion about this study.

HO have get in on a discussion about this study.

SH have get in on a discussion about this study.

TS have get in on a discussion about this study.

YS have given final approval of the version to be published.

All authors read and approved the final manuscript.

## Ethical approval and patient consent

This study was approved (approval No. 13-B-344) by the Research Ethics Committee of the International University of Health and Welfare, Tochigi, Japan.

We obtained consent from both of the patients for publishing this case report.

## Funding sources

None to declare.

## Registration of Research Studies

In accordance with the Declaration of Helsinki 2013, all research involving human participants has to be registered in a publicly accessible database. Please enter the name of the registry and the unique identifying number (UIN) of your study.

You can register any type of research at http://www.researchregistry.com to obtain your UIN if you have not already registered. This is mandatory for human studies only. Trials and certain observational research can also be registered elsewhere such as: ClinicalTrials.gov or ISRCTN or numerous other registries.

## Provenance and peer review

Not commissioned, externally peer reviewed.

## Declaration of competing interest

The authors declare no conflicts of interest including the manufacturer of the clips.

The authors were not involved at all in manufacture of the clips.
